# Taxonomic classification for microbiome analysis, which correlates well with the metabolite milieu of the gut

**DOI:** 10.1186/s12866-018-1311-8

**Published:** 2018-11-16

**Authors:** Yoshihisa Wakita, Yumi Shimomura, Yusuke Kitada, Hiroyuki Yamamoto, Yoshiaki Ohashi, Mitsuharu Matsumoto

**Affiliations:** 1 Frontier Laboratories for Value Creation, Sapporo Holdings Ltd., Yaizu, Shizuoka, 425-0013 Japan; 2 Dairy Science and Technology Institute, Kyodo Milk Industry Co. Ltd., Hinode-machi, Tokyo, 190-0182 Japan; 3 Human Metabolome Technologies, Inc., Tsuruoka, Yamagata, 997-0052 Japan

**Keywords:** Gut microbiome, Metabolome, Taxonomy, 16S amplicon sequencing, Principal component analysis

## Abstract

**Background:**

16S rRNA gene amplicon sequencing analysis (16S amplicon sequencing) has provided considerable information regarding the ecology of the intestinal microbiome. Recently, metabolomics has been used for investigating the crosstalk between the intestinal microbiome and the host via metabolites. In the present study, we determined the accuracy with which 16S rRNA gene data at different classification levels correspond to the metabolome data for an in-depth understanding of the intestinal environment.

**Results:**

Over 200 metabolites were identified using capillary electrophoresis and time-of-flight mass spectrometry (CE-TOFMS)-based metabolomics in the feces of antibiotic-treated and untreated mice. 16S amplicon sequencing, followed by principal component analysis (PCA) of the intestinal microbiome at each taxonomic rank, revealed differences between the antibiotic-treated and untreated groups in the first principal component in the family-, genus, and species-level analyses. These differences were similar to those observed in the PCA of the metabolome. Furthermore, a strong correlation between principal component (PC) scores of the metabolome and microbiome was observed in family-, genus-, and species-level analyses.

**Conclusions:**

Lower taxonomic ranks such as family, genus, or species are preferable for 16S amplicon sequencing to investigate the correlation between the microbiome and metabolome. The correlation of PC scores between the microbiome and metabolome at lower taxonomic levels yield a simple method of integrating different “-omics” data, which provides insights regarding crosstalk between the intestinal microbiome and the host.

**Electronic supplementary material:**

The online version of this article (10.1186/s12866-018-1311-8) contains supplementary material, which is available to authorized users.

## Background

The metabolic system of the microbiome is intricate, and the microbiome exerts a major influence on the host via the metabolome. A previous study comparing the colonic metabolome between germ-free (GF) and ex-GF mice harboring intestinal microbiota from specific pathogen-free mice showed that the intestinal microbiome strongly influenced the low-molecular-weight metabolites in the colonic lumen [1]. Few studies have elucidated the relationship between the metabolome produced by the intestinal microbiome and the host, although metabolomic studies on intestinal microbiome have increased and the association of the metabolome with the host have been suggested [2]. Only few specific metabolites produced by the intestinal microbiome have been reported to possess bioactive functions; for example, acetic acid improves the barrier function of intestinal epithelial cells [3], and butyric acid influences the differentiation of Treg cells [4].

With significant advancements in DNA sequencing technology, metagenomic analysis [5, 6] and 16S rRNA gene amplicon sequencing analysis (16S amplicon sequencing) [7, 8] have been developed over the last decade, which allow comprehensive phylogenetic assessment of the intestinal microbiome. Assessment of the human intestinal microbiome has revealed that approximately 1200 species inhabit the human intestine and that individual differences are significantly large [9]. Ecological studies on the intestinal microbiome have elucidated the relation between the *Firmicutes*/*Bacteroidetes* ratio and obesity [10] and revealed the existence of enterotypes among countries [11]. The former study used phylum-level analysis, whereas the latter used genus-level analysis. Furthermore, microbiome researchers are now using database-independent operational taxonomic unit (OTU)-based methods [12–14]. Hence, most microbiologists use wide taxonomic levels for studies that encompass comprehensive intestinal microbiome analyses and discuss the effects of the intestinal microbiome on the host [7, 9–11, 14–26]. However, reliable reports describing the accuracy with which the 16S amplicon sequencing data at different taxonomic classification levels correspond to metabolome data are lacking.

Under these circumstances, we attempted to determine appropriate classification levels to understand the relationship between the intestinal microbiome and metabolome using 16S amplicon sequencing. A decision regarding the appropriate levels of taxonomic classification that correlate with the differences in the metabolome will contribute to a detailed understanding of the crosstalk between the intestinal environment and host health. In this study, we analyzed feces derived from antibiotic-treated mice and untreated mice using 16S amplicon sequencing and metabolomics. These two omics data were analyzed using principal component analysis (PCA) to identify the appropriate taxonomic classification levels. In addition, we attempted to integrate the PCA of both omics data to ascertain the relationship between specific metabolites and bacteria.

## Results

### Fecal metabolome

**Fig. 1:**
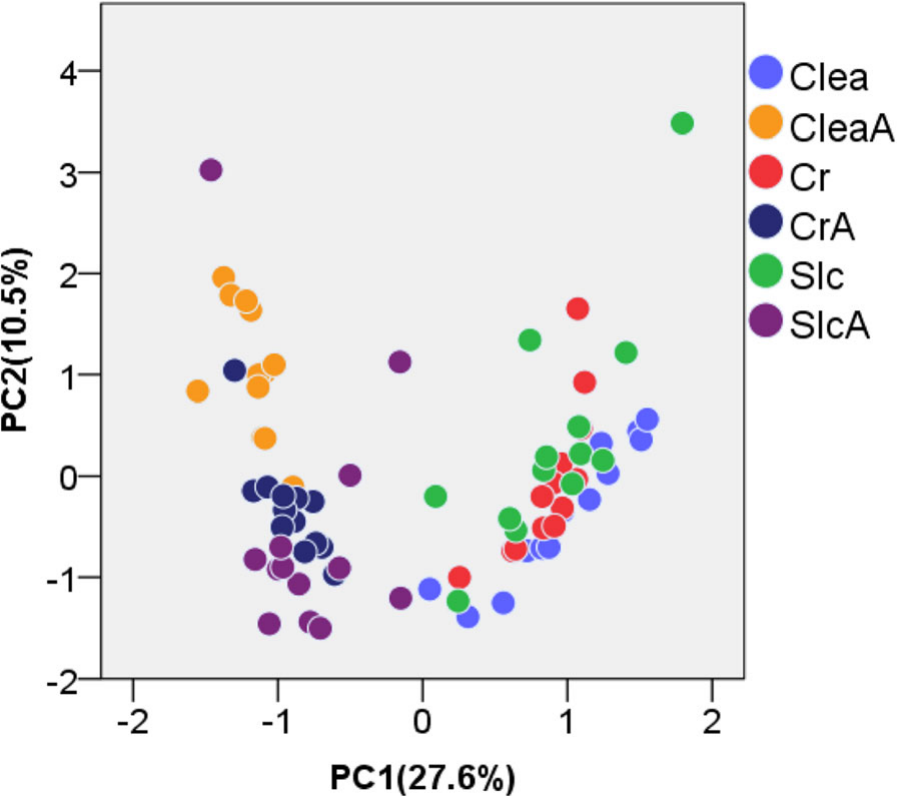
Principal component analysis (PCA) of the profiling data from the intestinal metabolome. C57BL/6 mice from three breeders were divided into two groups: untreated mice (Clea, Charles river (Cr), and Slc) and antibiotic-treated mice (CleaA, CrA, and SlcA). CE-TOFMS-based metabolomics was performed

>In this study, mice purchased from three breeders were divided into two groups (antibiotic-treated group and untreated group) and analyzed. The purpose of using antibiotics was to mimic a dysbiosis-like intestinal environment with less diverse microbiome, which is a significantly negative alteration of the composition and function of the gut microbiome. In addition, mice from three different sources were used to obtain data from the normal intestinal microbiome, in which the differences between individuals were smaller than those between intestinal microbiomes treated and not treated with antibiotics. Capillary electrophoresis and time-of-flight mass spectrometry (CE-TOFMS) identified 174, 154, 164, 148, 162, and 176 metabolites from the fecal metabolome of untreated mice obtained from Clea Japan Inc. (Clea), Charles River Laboratories Japan, Inc. (Cr), and Japan SLC Inc. (Slc), as well as their antibiotic-treated counterparts (CleaA, CrA, and SlcA, respectively); 205 metabolites were identified from the combined sample (Additional file 1). The PCA results showed that the metabolic profile was clearly divided into two groups—antibiotic-treated mice and untreated mice—based on PC1 scores (Fig. 1). In contrast, there were no clear differences among breeders between untreated mice, although CleaA clusters were observed among antibiotic-treated mice based on PC2 scores. Forty-one (~ 20%) out of 205 metabolites were selected when the absolute value of principal component loading (PCL) was > 0.7 (Additional file 2). One hundred and twenty-one (~ 60%) metabolites were selected when the absolute value of PCL was > 0.4. Statistical hypothesis testing of the PCL in PC1 was performed, and the correlation between the PC1 score and each metabolite level was observed to be statistically significant at *p* < 2.43 × 10^− 4^. The relative area of metabolites with an absolute PCL value > 0.7 is shown in Additional files 3 and 4.

### Fecal microbiome

**Fig. 2:**
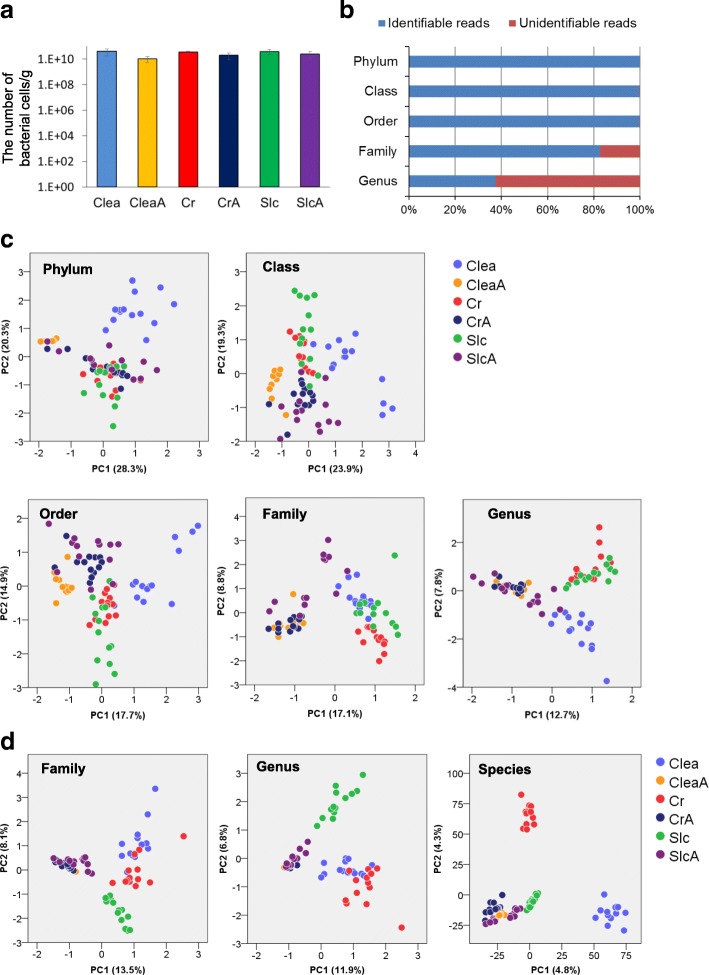
PCA of the profiling data from the intestinal microbiome. Microbiome analysis was performed using amplicon sequencing of the V1 and V2 regions of the 16S rRNA gene. Quantification of total bacterial was performed using real-time PCR. **a** Total bacterial numbers calculated using real-time PCR. **b** Rates of classifiable reads by RDP classifier. **c** PCA of classification data by RDP classifier. Differences between the antibiotic-treated and untreated groups in PC1 were recognized in the family- and genus-level analyses, although this was not recognized in the case of phylum, order, or class (**d**) PCA of family-, genus-, and species-level classification data after OTU-based analysis

There were no remarkable differences between total bacterial numbers per g of feces for the 6 groups (Fig. 2a). Therefore, on the 4th day after the antibiotic treatment was stopped, the effects of antibiotics on cell numbers of the intestinal microbiome were limited. In total, 1,838,586 reads (13,943–35,878 reads per sample) were obtained by 16S amplicon sequencing and selection using the QIIME pipeline. Blank data such as “g_” were interpreted as unidentifiable data due to the possibility that no-named OTUs are assigned same genus despite including several unknown genera. The identification percentages (the number of identified reads/the total number of reads) of each taxonomic level—phylum, class, order, family, and genus—were 99.9%, 99.8%, 99.8%, 82.9%, and 37.5%, respectively (Fig. 2b). The low percentage of identified bacterial genera was probably because of the dearth of identified bacteria in mice intestinal microbiome compared to that in the human microbiome [27]. Moreover, the percentage of assignable reads at the genus level was 82.9% including data pertaining to blanks, which was similar to identification percentage at the family level. Nine phyla, 15 classes, 22 orders, 40 families, and 59 genera were detected (Additional files 5, 6, 7, 8 and 9). Differences between the antibiotic-treated and untreated groups in PC1 were recognized in the family- and genus-level analyses of the PCA result of each taxonomic rank (Fig. 2c). In the untreated group, subclusters, which reflected each breeder, were recognized in PC2 in the case of genus- or family-level analyses, although these subclusters were not observed in the metabolome PCA. There was no difference between groups in the antibiotic-treated mice.

Owing to the lower identification percentage of families and genera than that of phyla, classes, and orders (Fig. 2b), the OTU-based classification was investigated at the family and genus levels according to a previously described method [28]. In addition, OTU-level (99% similarity analysis, i.e. species-level) analysis was performed to investigate the whole microbiome structure, including the unidentified bacteria at the level of family and genus using the RDP classifier. The value of similarity at the species level classification of OTUs was decided by referring to a previous study by Kim et al. [29]. The similarity of the 16S rRNA gene sequences in the OTUs that were classified as different families within the same order was 71.3% (Additional files 10 and 11). The similarity among 16S rRNA gene sequences in the OTUs that were classified as different genera within the same family was 77.1% (Additional files 11, 12 and 13). OTU-based family, genus, and species-level classification resulted in 76, 318, and 21,966 OTUs, respectively. Species level distribution of OTUs among the 6 groups is shown in Additional file 14. When the results of the PCA were assessed based on proportions of these OTUs, differences between antibiotic-treated and untreated groups were recognized in PC1 (Fig. 2d) for family, genus, and species-level classification. These differences at family and genus-level analysis were similar to those obtained from the PCA based on RDP data. In the untreated mouse group, subclusters that reflected each breeder were recognized in PC2, similar to the PCA of RDP data.

**Fig. 3:**
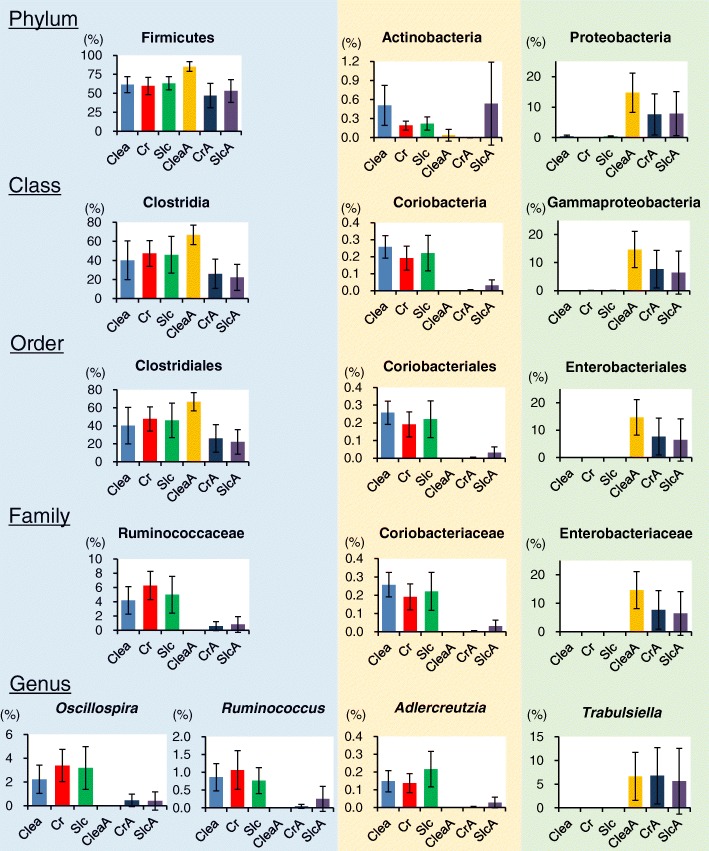
Relative abundance of genera with an absolute value of PCL > 0.7 for PC1 and higher taxonomic ranks of these genera. Data represent mean ± SD. Levels of the genera *Ruminococcus* and *Oscillospira* in the untreated group were higher than those in the antibiotic-treated group. Members of the family *Ruminococcaceae* were similarly represented in the two groups. However, this similarity was not observed at the level of phylum (*Firmicutes*), class (*Clostridia*), or order (*Clostridiales*). Levels of the genus *Adlercreutzia* in the untreated group were higher than in the antibiotic-treated group, and this trend continued till the class level (*Coriobacteriia*). Levels of the genus *Trabulsiella* in the antibiotic-treated group were higher than that in the untreated group, and this trend continued till the phylum level (*Proteobacteria*)

Four (10%) of 40 families and four (about 7%) of 59 genera were identified using an absolute PCL value of > 0.7 for each PC1 (Additional file 15). Fourteen (35%) families and 12 (about 20%) genera were identified using an absolute PCL value of > 0.4. Statistical hypothesis testing of the PCL in PC1 was performed, and these correlations in families and genera were statistically significant at *p* < 1.25 × 10^− 3^ and 8.47 × 10^− 4^, respectively. Relative abundance of the genera (the lowest taxonomic rank detected by RDP) with an absolute value of PCL > 0.7 for PC1 and higher taxonomic ranks of these genera are shown in Fig. 3. Levels of the genera *Ruminococcus* and *Oscillospira* in the untreated group were higher than those in the antibiotic-treated group. The family *Ruminococcaceae* and the higher taxonomic ranks of these genera were similar between the two groups. However, this similarity was not observed at the level of phylum (*Firmicutes*), class (*Clostridia*), or order (*Clostridiales*). On the contrary, levels of the genus *Adlercreutzia* in the untreated group were higher than that in the antibiotic-treated group, and this trend continued till the class level (*Coriobacteriia*). Levels of the genus *Trabulsiella* in the antibiotic-treated group were higher than those in the untreated group, and this trend continued till the phylum level (*Proteobacteria*).

### Correlation between the PC1 score of the metabolome and microbiome

**Fig. 4:**
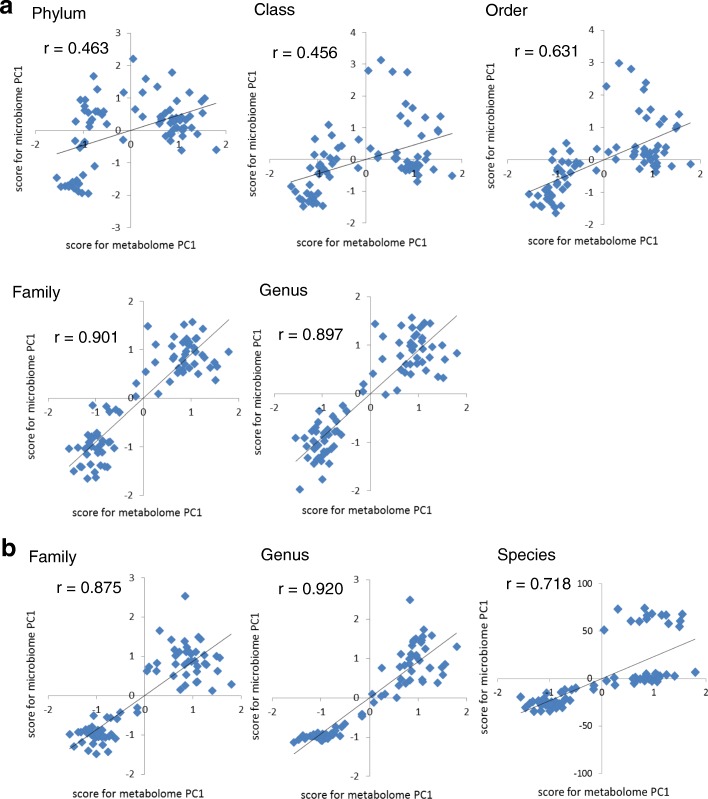
Correlation between the PC1 score of the metabolome and that of the microbiome. Scores for each principal component 1 (PC1) of the metabolome and microbiome were plotted in the X-Y axis, followed by calculation of Pearson’s product-moment correlation coefficient. **a** PC1 of classification data by RDP classifier. At the genus and family levels, a stronger correlation (*r* > 0.7) between the PC1 of the metabolome and microbiome was observed compared to the case of phylum, order, or class. **b** PC1 of family-, genus-, and species-level classification data after OTU-based analysis

Figure 4 shows the X-Y axis plots of the PC1 score for the metabolome and microbiome. The correlation coefficients at phylum, class, order, family, and genus level using RDP data were 0.463 (*p* = 1.98 × 10^− 5^), 0.456 (*p* = 2.76 × 10^− 5^), 0.631 (*p* = 5.94 × 10^− 10^), 0.901 (*p* = 3.26 × 10^− 29^), and 0.897 (*p* = 1.33 × 10^− 28^), respectively (Fig. 4a). At the genus and family levels, a strong correlation (*r* > 0.7) between the PC1 of the metabolome and microbiome was observed. In addition, the correlation coefficients at the levels of family, genus and species using OTU-based classification were 0.875 (*p* = 1.16 × 10^− 25^), 0.920 (*p* = 1.40 × 10^− 32^), and 0.718 (*p* = 1.38 × 10^− 13^) (Fig. 4b), respectively, showing results similar to those obtained from RDP classification.

### Correlation between metabolite concentration and relative abundance of the microbiome

**Fig. 5:**
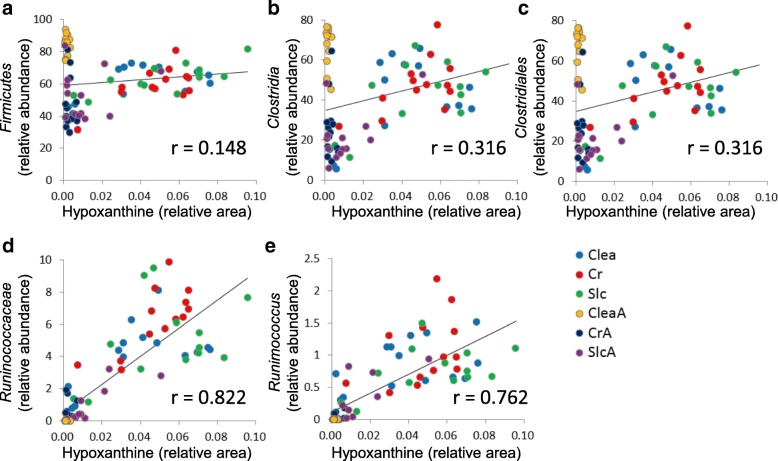
Correlation between hypoxanthine and each taxonomic classification rank. With respect to hypoxanthine, for which the principal component loading for the metabolome PC1 score was high (0.893), correlations between the relative area of hypoxanthine and the relative abundance of the genus *Ruminococcus* and its higher taxonomic class (family *Ruminococcaceae*, order *Clostridiales*, class *Clostridia*, phylum *Firmicutes*) were compared. The correlation coefficients at (**a**) phylum-, (**b**) class-, (**c**) order-, (**d**) family-, and (**e**) genus-levels were 0.148 (*p* = 0.20), 0.316 (*p* = 4.81 × 10^− 3^), 0.316 (*p* = 4.85 × 10^− 3^), 0.822 (*p* = 2.71 × 10^− 20^), and 0.762 (*p* = 5.09 × 10^− 16^), respectively

The level of correlation between taxa at different taxonomic levels and metabolites is summarized in Table 1. As the taxonomic level descended from phylum to genus, the proportion of metabolites correlating to any of the taxa increased. In contrast, the proportion of taxa correlating to any of the metabolites remained almost unchanged. An overall relation between metabolite and microbiome was evaluated using heat maps based on the correlation coefficient between metabolite concentration and relative abundance of the microbiome at different taxonomic ranks (Additional file 16). We observed that several bacterial groups, for example, *Ruminococcaceae, Coriobacteriaceae, Enterobacteriaceae*, and *Enterococcaceae* at the family level correlated to several metabolites. Subsequently, metabolites that were strongly (*r* > 0.7 or *r* < − 0.7) influenced by these families were selected (Additional file 17). From this list, we focused on the correlation between the relative area of hypoxanthine and relative abundance of the family *Ruminococcaceae* and its lower and higher taxonomic ranks (genus *Ruminococcus*, order *Clostridiales*, class *Clostridia*, phylum *Firmicutes*) (Fig. 5). Strong correlations (r > 0.7) between hypoxanthine concentration and the abundance of *Ruminococcaceae* (family) or *Ruminococcus* (genus) were observed.

**Table 1:** Level of correlation between taxa at different taxonomic rank and the metabolites

	Taxonomic rank					
	Phylum	Class	Order	Family	Genus	Species^c^
Number of metabolites^a^	10 (4.9%)	21 (10.2%)	22 (10.7%)	51 (24.9%)	44 (21.5%)	121 (59.0%)
Number of taxa^b^	4/9 (44.4%)	6/15 (40.0%)	8/22 (36.4%)	16/40 (40.0%)	19/59 (32.2%)	2889/21,966 (13.2%)

^a^No. of metabolites which correlate to the any of taxa (*r* > 0.7)

^b^No. of taxa which correate to the any of metabolites (*r* > 0.7) / No. of taxa observed

^c^Species level classification of OTUs

## Discussion

PCA is frequently used to analyze both the fecal metabolome [1, 30, 31] and microbiome [7, 15, 16, 19, 20]. In this study, we searched for the taxonomic classification level of the intestinal microbiome that correlated with the metabolome profile using PCA. This study is also an example of simple integrated omics for interpreting omics data from the fecal metabolome and microbiome.

The PCA profiles of 16S amplicon sequencing were similar to those of the metabolomics analysis at the family and genus levels, but not at the phylum, class, or order levels, indicating that family and genus are the appropriate taxonomic levels to study the relationship between the intestinal microbiome and the host’s health using 16S amplicon sequencing. The results of the level of correlation between the taxa observed at the different taxonomic levels and the metabolites (Table 1) supported this observation. Considering the phylogenetic evolution of bacteria, it can be assumed that the phylogenies of bacteria branch out as the taxonomic level is segmented from phylum to species, and each phylogeny-divided bacterial group possesses a particular metabolic pathway. To the best of our knowledge, this is the first study to demonstrate that family, genus, or species are more strongly associated with the metabolome than higher taxonomic levels. The phylum *Firmicutes* and its class *Clostridia* include a variety of bacterial genera. This is probably one of the reasons why correlation between the metabolome and microbiome in the analysis conducted at the levels of order, class, and phylum was low. This is supported by the results shown in Fig. 3.

Although the PC2 scores of the metabolome and 16S amplicon sequencing-based microbiome partially reflected the differences in breeder (Figs. 1, 2c, and d), obvious correlation was not observed for the PC2 of the metabolome and microbiome at any taxonomic level (see Additional file 18). The extent of difference in the microbiome between each group in untreated mice was higher than that in the metabolome, indicating that a difference in the microbiome under normal conditions, which is smaller than that under dysbiosis, does not significantly influence the metabolome. In contrast, despite no obvious differences in the microbiome in the antibiotic-treated mice, a subcluster of CleaA in the metabolome was observed based on PC2, indicating that the intestinal metabolome might be influenced by the host, including by intestinal absorption, in mice with dysbiosis. The metabolome is not only a product of bacteria but also of co-metabolic pathways between the host and bacteria. In this regard, not only the microbiome, but also the host’s condition, can be used to investigate the crosstalk between the microbiome and host via the metabolome.

The correlations of PC1 between the metabolome and microbiome at the family and genus levels suggest a strong likelihood that both key metabolites and bacteria, which play important roles in the metabolism of the gut microbiome, can be detected by integrating PCA data from the fecal metabolome and the 16S amplicon sequencing-based microbiome. In fact, we observed correlations between hypoxanthine and the genus *Ruminococcus* and family *Ruminococcaceae*. Figure 5 reveals that the regression line passed near the origin, indicating that the hypoxanthine concentration depends on the numbers of these bacteria. Furthermore, the levels of fatty acids and nucleotide catabolites in untreated mice were higher than those in the antibiotic-treated mice (Additional file 3). The relative abundance of the families *Ruminococcaceae* and *Coriobacteriaceae* was high, but that of the families *Enterobacteriaceae* and *Enterococcaceae* was low (Additional file 8), suggesting that the presence of fatty acids and nucleotide catabolites is influenced by these families. It is interesting that *Ruminococcus*, which belongs to the family *Ruminococcaceae*, is a key player in the degradation of resistant starch and butyric acid production [32, 33]. Interestingly, hypoxanthine, spermidine, and 4-guanidinobutyric acid, which have been reported to be beneficial or toxic for mammalian cells, were affected by the antibiotic-treatment, and the relative area of each of these metabolites was related to the relative abundance of the family *Ruminococcaceae*. Hypoxanthine modulates energy metabolism in intestinal epithelial cells and is critical for intestinal barrier function [34]. Spermidine is known to reinforce the intestinal mucosal barrier function [35] and promote autophagy [36]. Interestingly, the genus *Ruminococcus* has been suggested to be associated with polyamine production in humans [37]. It is noteworthy that the known relationship between intestinal bacteria and bioactive metabolites was observed in our study by integrating the PCA data. 4-Guanidinobutyric acid causes convulsive toxicity [38]. In addition, 4-guanidinobutyric acid level correlated positively with the families *Enterococcaceae* and *Enterobacteriaceae* (Additional file 17). Increasing levels of these families have been reported to be an indication of dysbiosis [39, 40]. Our results indicated the possibility that *Enterococcaceae* and *Enterobacteriaceae* are involved in 4-guanidinobutyric acid production, thereby contributing to the adverse effect of dysbiosis.

In summary, we suggest that lower taxonomic levels such as family, genus, or species are useful for investigating the crosstalk between the intestinal microbiome and the host based on the results of 16S amplicon sequencing. We expect that new information regarding the relationship between the intestinal conditions and host will be revealed in the future by combining metabolome and lower taxonomic level microbiome analysis. In addition, we demonstrated that the novel method of integrated omics successfully represented the intestinal conditions by integrating the PCA data from the fecal metabolome and 16S amplicon sequencing of the microbiome.

## Conclusions

Lower taxonomic ranks such as family, genus, or species are preferable for 16S amplicon sequencing to investigate the correlation between the microbiome and metabolome. The correlations of PC scores between the microbiome and metabolome at the family-, genus-, and species-levels provide a simple method of integrating different “-omics” data, which can elucidate the crosstalk between the intestinal microbiome and host.

## Methods

### Mice

Eight-week-old male C57BL/6 mice were purchased from Clea Japan Inc. (Tokyo, Japan), Japan Charles River Inc. (Yokohama, Japan), and Japan SLC Inc. (Shizuoka, Japan). These mice were bred at the Dairy Science and Technology Institute, Kyodo Milk Industry, Co. Ltd., Tokyo, Japan. Immediately after their arrival, mice from each breeder were divided into two groups: untreated mice [Clea: *n* = 14, Charles River (Cr): *n* = 13, and Slc: *n* = 13] and antibiotic-treated mice (CleaA: *n* = 12, CrA: *n* = 13, and SlcA: *n* = 13). Six to seven animals were housed per cage (depth, 41 cm; width, 26 cm; height, 21 cm) and were provided water and commercial CL-2 pellets (Clea Japan, Inc.) ad libitum. After 5 days of preliminary breeding, the antibiotic-treated mice were administered water containing antibiotics (1 g/L ampicillin, 1 g/L neomycin, 1 g/L metronidazole, and 0.5 g/L vancomycin) ad libitum for 3 days, which have been reported to be sufficient for depleting almost all detectable commensal bacteria [41]. Feces from the antibiotic-treated mice were collected on the 4th day after the antibiotic treatment, and that from the untreated group were collected at the same time. The protocols were approved by the Kyodo Milk Animal Use Committee (permit number: 2013–01). All the experimental procedures were performed according to the guidelines of the Animal Care Committee of Kyodo Milk Industry Co. Ltd., and were in accordance with the Guide for the Care and Use of Laboratory Animals published by the National Academies Press.

### Preparation of the fecal metabolome

Fresh samples (approximately 100 mg) were diluted nine-fold using Dulbecco’s phosphate-buffered saline (D-PBS; Gibco, Palo Alto, CA, USA) and extracted thrice by intense mixing for 1 min and resting for 5 min on ice. The upper aqueous portion, without the precipitate at the bottom, was collected and centrifuged (12,000×*g* for 10 min at 4 °C) 1 min after the extraction, and 200 μL supernatant was centrifugally filtered through a 5 kDa cutoff filter (Ultrafree-MC; Millipore, Bedford, MA, USA). The filtrate was stored at − 80 °C until use.

### Capillary electrophoresis and time-of-flight mass spectrometry

The metabolomics measurement and data processing were performed as described previously using an Agilent capillary electrophoresis system (Santa Clara, CA, USA) [1] based on the method of Ooga et al. [42]. All processing was performed by personnel at Human Metabolome Technologies, Inc.

### Fecal bacterial DNA extraction

The precipitate obtained after the first centrifugation during the preparation of the fecal metabolome was used for microbiome analysis. This bacterial DNA was isolated using the methods described by Matsuki et al. [43] with some modifications [1].

### Microbiome analysis

The number of total bacteria was quantified using quantitative real-time PCR as described previously, with some modification [44]. Briefly, PCR was performed with a StepOne Real-Time PCR System (Applied Biosystems) using SYBR Premix Ex Taq II ROX Plus (Takara Bio Inc., Otsu, Japan), and the Total F (TCCTACGGGAGGCAGCAGT) and Total-R (GGACTACCAGGGTATCTAATCCTGTT) primers specific for total bacteria [45]. The DNA was extracted from *Bacteroides uniformis* JCM5828^T^, which is the most common bacterial species in the human microbiome [9], was used as the real-time PCR standard.

Primers for the amplification of the V1 and V2 regions of the 16S rRNA gene reported by Kim et al. [46] were used with some modifications. The following primers were used: forward primer (5′-CCATCTCATCCCTGCGTGTCTCCGACTCAGNNNNNNNNNNGTagrgtttgatymtggctcag-3′) containing the Ion PGM sequencing primer A-key, a unique error-correcting 10~ 12 bp barcode sequence (indicated by N), “GT” spacer, and 27Fmod (agrgtttgatymtggctcag); the reverse primer (5′-CCTCTCTATGGGCAGTCGGTGATtgctgcctcccgtaggagt-3′) contained the Ion PGM primer P1 and 338R (tgctgcctcccgtaggagt). PCR was performed in a 25 μL reaction volume. Each reaction mixture contained 22.5 μL platinum PCR mix, 2 μL template DNA (~ 4 ng), and 0.5 μL 10 μM primer mix. The amplification reaction was performed in a Veriti thermal cycler (Applied Biosystems, Foster City, CA, USA) using the following program: 3 min at 94 °C, followed by 25 cycles of 30 s each at 94 °C, 45 s at 55 °C, and 1 min at 68 °C. After each reaction, the mixture was purified using PureLink Quick PCR purification kit (Invitrogen, Carlsbad, CA, USA). The concentration of each purified sample was measured using the Qubit 2.0 fluorometer (Life Technologies, Carlsbad, CA, USA). Purified samples were mixed at equal concentrations. The mixed sample was visualized by electrophoresis on a 2% agarose gel and purified by gel extraction using the FastGene Gel/PCR extraction kit (Nippon Genetics Co. Ltd., Tokyo, Japan). Subsequently, emulsion PCR and sequencing were performed using Ion PGM sequencing (Life Technologies). All sequence data were deposited in the DDBJ sequence read archive database under accession number DRA004549.

After sequencing, the obtained reads were analyzed using the QIIME pipeline (http://qiime.org/) [13] for taxonomic classification. The reads, which included precise primer sequences (27Fmod and 338R), were selected, and those with an average quality value > 20 were used for further analysis. The reads were grouped into OTUs with a sequence identity threshold of 97%, and chimeric OTUs were removed using ChimeraSlayer. The proportion of the intestinal microbiome at each taxonomic rank, such as phylum, order, class, family, and genus, was determined using the RDP classifier and the greengenes database (gg_13_8_otus / taxonomy / 97_otu_taxonomy).

On the basis of the OTUs and classification obtained from RDP, the distances among OTUs, which classified different families or genera among the same orders or families, respectively, were calculated using Mothur [47]. The average of each distance was used to set the cutoff parameter for differentiating among families or genera. Next, the OTUs that reflected the family or genus were obtained from representative sequences using an average neighbor algorithm. The 99% identity threshold was used for species-level analysis. Singleton OTUs were excluded for OTU-based classification.

### Statistical analysis

PCA of the metabolome (peak area of each metabolite) and intestinal microbiome (relative abundance of each taxonomic rank) data was performed with auto scaling using SPSS Statistics version 22 (IBM, North Castle, NY, USA) and/or R statistical software ver.3.4.2. Statistical hypothesis testing of the PCL in PCA was performed using Excel 2010 (Microsoft, Redmond, WA, USA) based on the R package mseapca described elsewhere [48]. The significance level was corrected for multiple comparisons using Bonferroni’s correction. The threshold for statistical significance in the metabolome was set at *p* < 2.44 × 10^− 4^. The thresholds for statistical significance in microbiomes using family and genus were set at *p* < 1.25 × 10^− 3^ and *p* < 8.47 × 10^− 4^, respectively.

(Significance level ÷ n (number of metabolites) = 0.05 ÷ 205 = 0.0002439)

(Significance level ÷ n (number of families) = 0.05 ÷ 40 = 0.00125)

(Significance level ÷ n (number of genera) = 0.05 ÷ 59 = 0.0008474)

Scores for each principal component 1 (PC1) of the metabolome and microbiome were plotted on the X- and Y-axis, respectively, followed by calculation of Pearson’s product-moment correlation coefficient using SPSS statistics. Pearson’s correlation coefficient for hypoxanthine and the microbiome was also calculated. The level of correlation between the taxa observed at the different taxonomic levels and the metabolites was evaluated using Pearson’s correlation.

## Additional files

Additional file 1: Relative peak area of metabolite by CE-TOFMS based metabolomics. (XLSX 75 kb)

Additional file 2: Principal component loading of each metabolite for metabolome first principal component (PC1). (XLSX 17 kb)

Additional file 3: Comparison of relative peak area of metabolite picked up by the PCL > 0.7. (DOCX 229 kb)

Additional file 4: Comparison of relative peak area of metabolite picked up by the PCL < − 0.7. (DOCX 119 kb)

Additional file 5: Relative abundance of intestinal microbiome (phylum). (DOCX 20 kb)

Additional file 6: Relative abundance of intestinal microbiome (class). (DOCX 19 kb)

Additional file 7: Relative abundance of intestinal microbiome (order). (DOCX 20 kb)

Additional file 8: Relative abundance of intestinal microbiome (family). (DOCX 24 kb)

Additional file 9: Relative abundance of intestinal microbiome (genus). (DOCX 27 kb)

Additional file 10: Similarities in the OTUs that are classified as different families within the same order. (DOCX 20 kb)

Additional file 11: Results of similarity calculation for OTU-based family and genus-level classification. (DOCX 15.1 kb)

Additional file 12: Similarities in the OTUs that are classified as different genera within the same family. (DOCX 20 kb)

Additional file 13: Example of similarity calculation (results of calculation in the yellow area of Additional file 12). (XLSX 39 kb)

Additional file 14: Relative abundance of intestinal microbiome (species level classification of OTUs). (XLSX 2182 kb)

Additional file 15: Principal component loading of each genus and family for microbiome first principal component (PC1). (XLSX 11.9 kb)

Additional file 16: Heat maps based on the correlation between the relative abundance of the microbiome at different taxonomic ranks and the metabolome. (PDF 206 kb)

Additional file 17: Correlation coefficients between selected bacterial families and metabolome. (PDF 27 kb)

Additional file 18: Correlation between score of PC2 of the metabolome and that of the microbiome. (DOCX 278 kb)

## Abbreviations

16S amplicon sequencing: 16S rRNA gene amplicon sequencing analysis
CE-TOFMS: Capillary electrophoresis and time-of-flight mass spectrometry
Clea: Mice obtained from Clea Japan, Inc.
CleaA: Antibiotic-treated Clea mice
Cr: Mice obtained from Charles River Laboratories Japan, Inc
CrA: Antibiotic-treated Cr mice
OTUs: Operational taxonomic units (OTUs)
PC: Principal component
PCA: Principal component analysis
PCL: Principal component loading
Slc: Japan SLC, Inc.
SlcA: Antibiotic-treated Slc mice
