# Supplementary material for: Taxonomic classification for microbiome analysis, which correlates well with the metabolite milieu of the gut
Source: BMC Microbiol. 2018 Nov 16;18:188. doi: 10.1186/s12866-018-1311-8 (PMC6240276; doi:10.1186/s12866-018-1311-8)
Supplement: Supplementary file 4 — Comparison of relative peak area of metabolite picked up by the PCL < − 0.7. (DOCX 119 kb) [file 12866_2018_1311_MOESM4_ESM.docx]

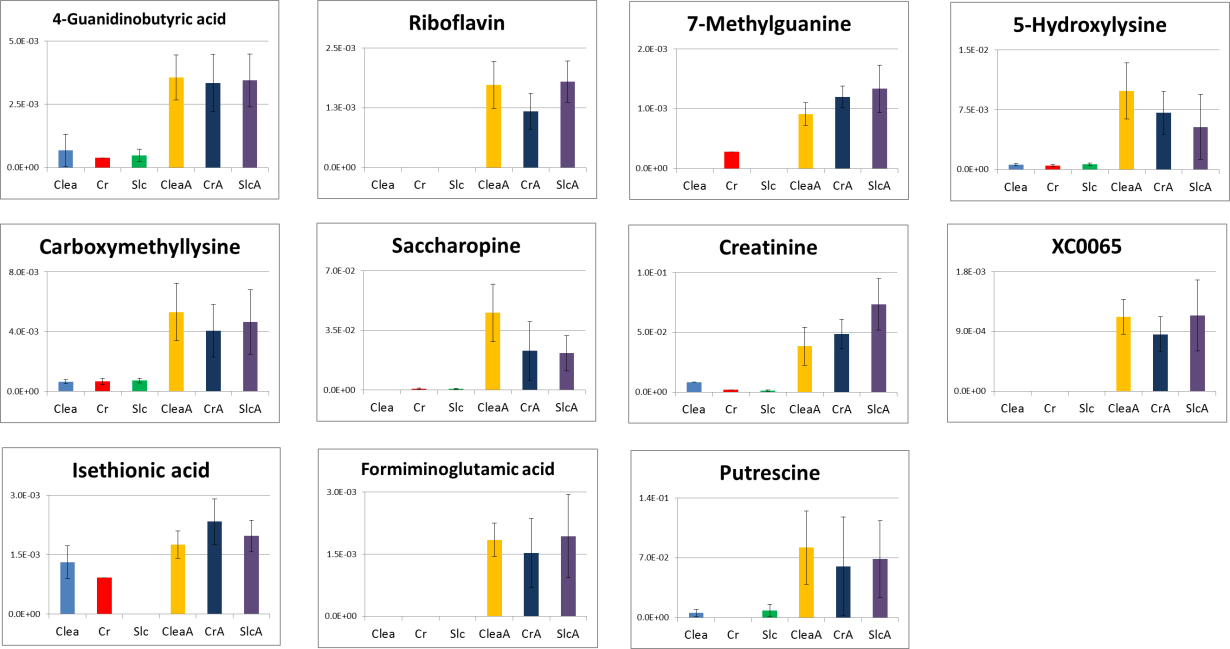


**Additional File 4. Comparison of relative peak area of metabolite picked up by the PCL < -0.7**

Clea: untreated mice group of Clea, Cr: untreated group of Charles river, Slc: untreated group of Slc, CleaA: antibiotic-treated group of Clea, CrA: antibiotic-treated group of Charles river, SlcA: antibiotic-treated group of Slc. Data are represented as mean ± SD.
