# Supplementary material for: Taxonomic classification for microbiome analysis, which correlates well with the metabolite milieu of the gut
Source: BMC Microbiol. 2018 Nov 16;18:188. doi: 10.1186/s12866-018-1311-8 (PMC6240276; doi:10.1186/s12866-018-1311-8)
Supplement: Supplementary file 9 — Relative abundance of intestinal microbiome (genus). (DOCX 27 kb) [file 12866_2018_1311_MOESM9_ESM.docx]

**Additional File 9. Relative abundance of intestinal microbiome (genus)**
