# Supplementary material for: Taxonomic classification for microbiome analysis, which correlates well with the metabolite milieu of the gut
Source: BMC Microbiol. 2018 Nov 16;18:188. doi: 10.1186/s12866-018-1311-8 (PMC6240276; doi:10.1186/s12866-018-1311-8)
Supplement: Supplementary file 10 — Similarities in the OTUs that are classified as different families within the same order. (DOCX 20 kb) [file 12866_2018_1311_MOESM10_ESM.docx]

**Additional File 10. Similarities in the OTUs that are classified as different families within the same order**

Parentheses show the number of OTU.
