# Supplementary material for: Taxonomic classification for microbiome analysis, which correlates well with the metabolite milieu of the gut
Source: BMC Microbiol. 2018 Nov 16;18:188. doi: 10.1186/s12866-018-1311-8 (PMC6240276; doi:10.1186/s12866-018-1311-8)
Supplement: Supplementary file 11 — Results of similarity calculation for OTU-based family and genus-level classification. (DOCX 15.1 kb) [file 12866_2018_1311_MOESM11_ESM.docx]

**Additional File 11. Results of similarity calculation for OTU-based family and genus-level classification**
