# Supplementary material for: Taxonomic classification for microbiome analysis, which correlates well with the metabolite milieu of the gut
Source: BMC Microbiol. 2018 Nov 16;18:188. doi: 10.1186/s12866-018-1311-8 (PMC6240276; doi:10.1186/s12866-018-1311-8)
Supplement: Supplementary file 12 — Similarities in the OTUs that are classified as different genera within the same family. (DOCX 20 kb) [file 12866_2018_1311_MOESM12_ESM.docx]

**Additional File 12. Similarities in the OTUs that are classified as different genera within the same family**

Parentheses show the number of OUT. The yellow area shows the calculation value of Additional File 13.
