# Supplementary material for: Taxonomic classification for microbiome analysis, which correlates well with the metabolite milieu of the gut
Source: BMC Microbiol. 2018 Nov 16;18:188. doi: 10.1186/s12866-018-1311-8 (PMC6240276; doi:10.1186/s12866-018-1311-8)
Supplement: Supplementary file 16 — Heat maps based on the correlation between the relative abundance of the microbiome at different taxonomic ranks and the metabolome. (PDF 206 kb) [file 12866_2018_1311_MOESM16_ESM.pdf]

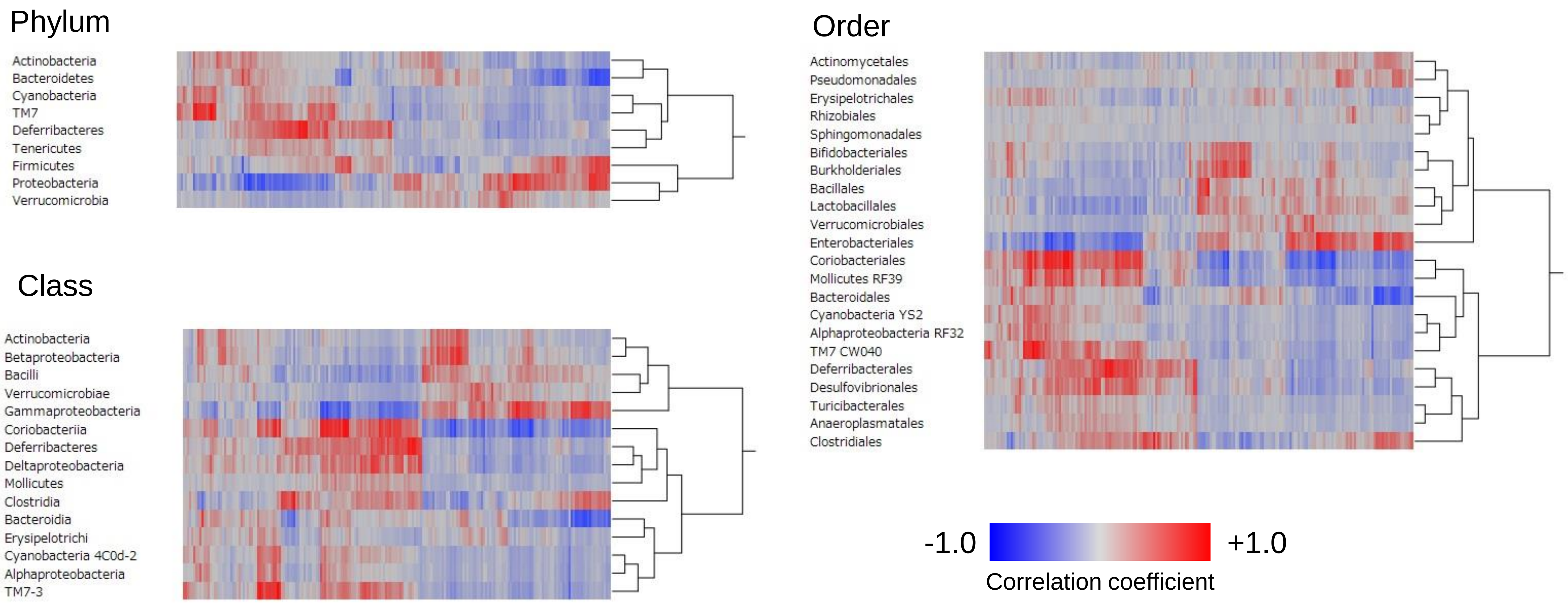

Additional File 16. Heat maps based on the correlation between the relative abundance of the microbiome at different taxonomic ranks and the metabolome

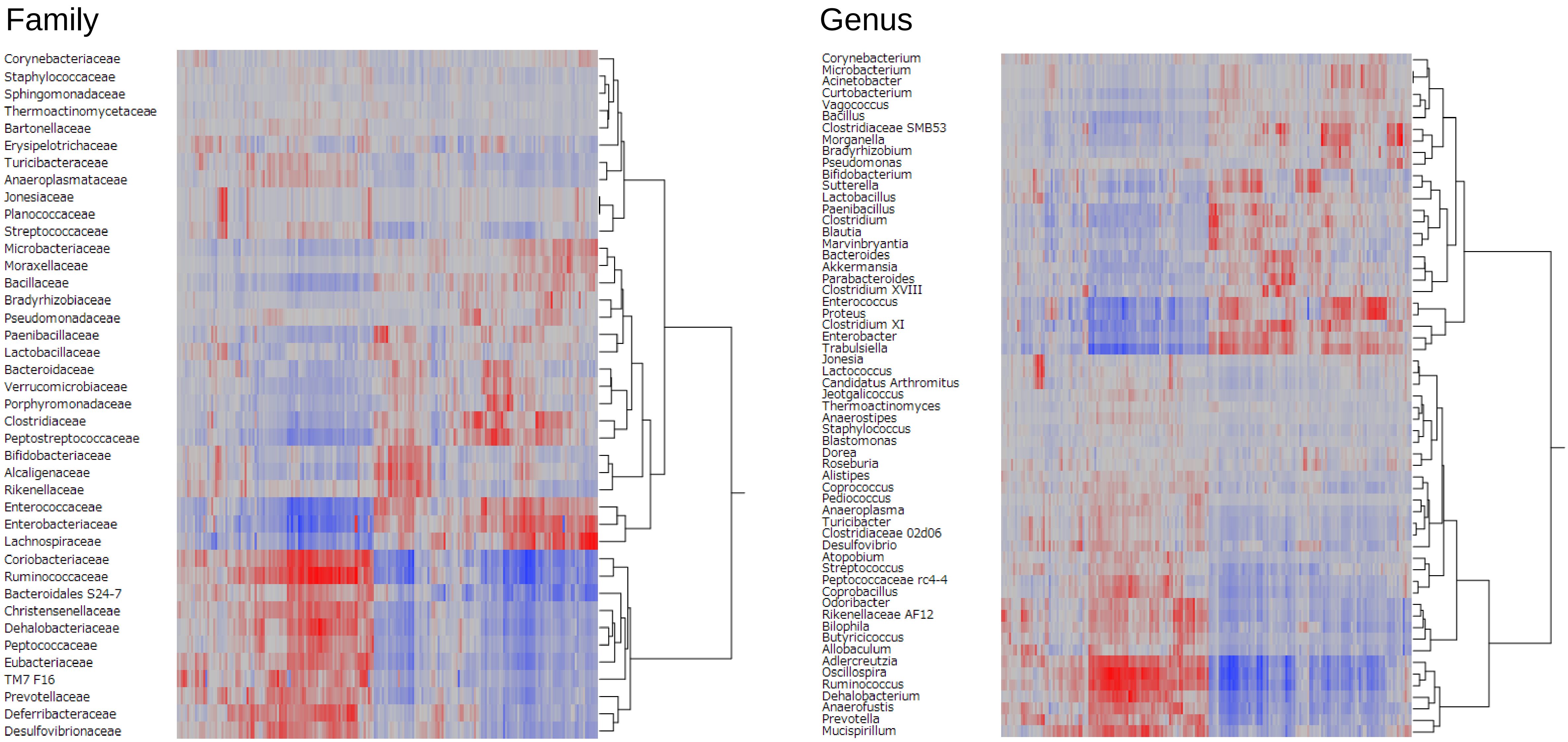

Additional file 16 (continued). Heat maps based on the correlation between the relative abundance of the microbiome at different taxonomic ranks and the metabolome
