# Supplementary material for: Taxonomic classification for microbiome analysis, which correlates well with the metabolite milieu of the gut
Source: BMC Microbiol. 2018 Nov 16;18:188. doi: 10.1186/s12866-018-1311-8 (PMC6240276; doi:10.1186/s12866-018-1311-8)
Supplement: Supplementary file 17 — Correlation coefficients between selected bacterial families and metabolome. (PDF 27 kb) [file 12866_2018_1311_MOESM17_ESM.pdf]

Additional File 17. Correlation coefficients between selected bacterial families and metabolome

	Ruminococcaceae	*Coriobacteriaceae*	*Enterococcaceae*	*Enterobacteriaceae*
Isovaleric acid	0.877	0.678	−0.525	0.701
N−Methylalanine	0.875	0.651	−0.503	−0.532
N−Acetylglucosamine 1−phosphate	0.868	0.607	−0.483	−0.510
Isobutyric acid	0.843	0.593	−0.471	−0.567
Pyridoxal	0.841	0.698	−0.596	−0.669
Dodecanedioic acid	0.840	0.749	−0.592	−0.573
Hypoxanthine	0.822	0.593	−0.528	−0.574
Cytidine	0.813	0.467	−0.470	−0.508
Glucose 1−phosphate	0.800	0.463	−0.420	−0.468
Uracil	0.793	0.584	−0.569	−0.563
Guanosine	0.792	0.509	−0.483	−0.522
XC0001	0.790	0.740	−0.532	−0.673
Glyceric acid	0.788	0.407	−0.419	−0.339
Guanine	0.783	0.615	−0.508	−0.578
Thiamine phosphate	0.774	0.448	−0.499	−0.516
Xanthine	0.767	0.614	−0.538	−0.577
Spermidine	0.765	0.601	−0.487	−0.563
1−Methyl−4−imidazoleacetic acid	0.759	0.766	−0.553	−0.594
Propionic acid	0.742	0.699	−0.616	−0.758
Azelaic acid	0.738	0.660	−0.571	−0.442
Inosine	0.727	0.450	−0.422	−0.456
XC0089	0.722	0.349	−0.351	−0.372
4−Pyridoxic acid	0.719	0.708	−0.676	−0.704
6−Aminohexanoic acid	0.639	0.710	−0.482	−0.515
3−(4−Hydroxyphenyl)propionic acid	0.620	0.702	−0.548	−0.669
XC0029	0.605	0.703	−0.454	−0.479
XC0017	−0.558	−0.579	0.725	0.505
Formiminoglutamic acid	−0.641	−0.639	0.666	0.719
7−Methylguanine	−0.694	−0.748	0.476	0.453
Riboflavin	−0.701	−0.733	0.557	0.661
4−Guanidinobutyric acid	−0.758	−0.773	0.494	0.560
