# Supplementary material for: Taxonomic classification for microbiome analysis, which correlates well with the metabolite milieu of the gut
Source: BMC Microbiol. 2018 Nov 16;18:188. doi: 10.1186/s12866-018-1311-8 (PMC6240276; doi:10.1186/s12866-018-1311-8)
Supplement: Supplementary file 18 — Correlation between score of PC2 of the metabolome and that of the microbiome. (DOCX 278 kb) [file 12866_2018_1311_MOESM18_ESM.docx]

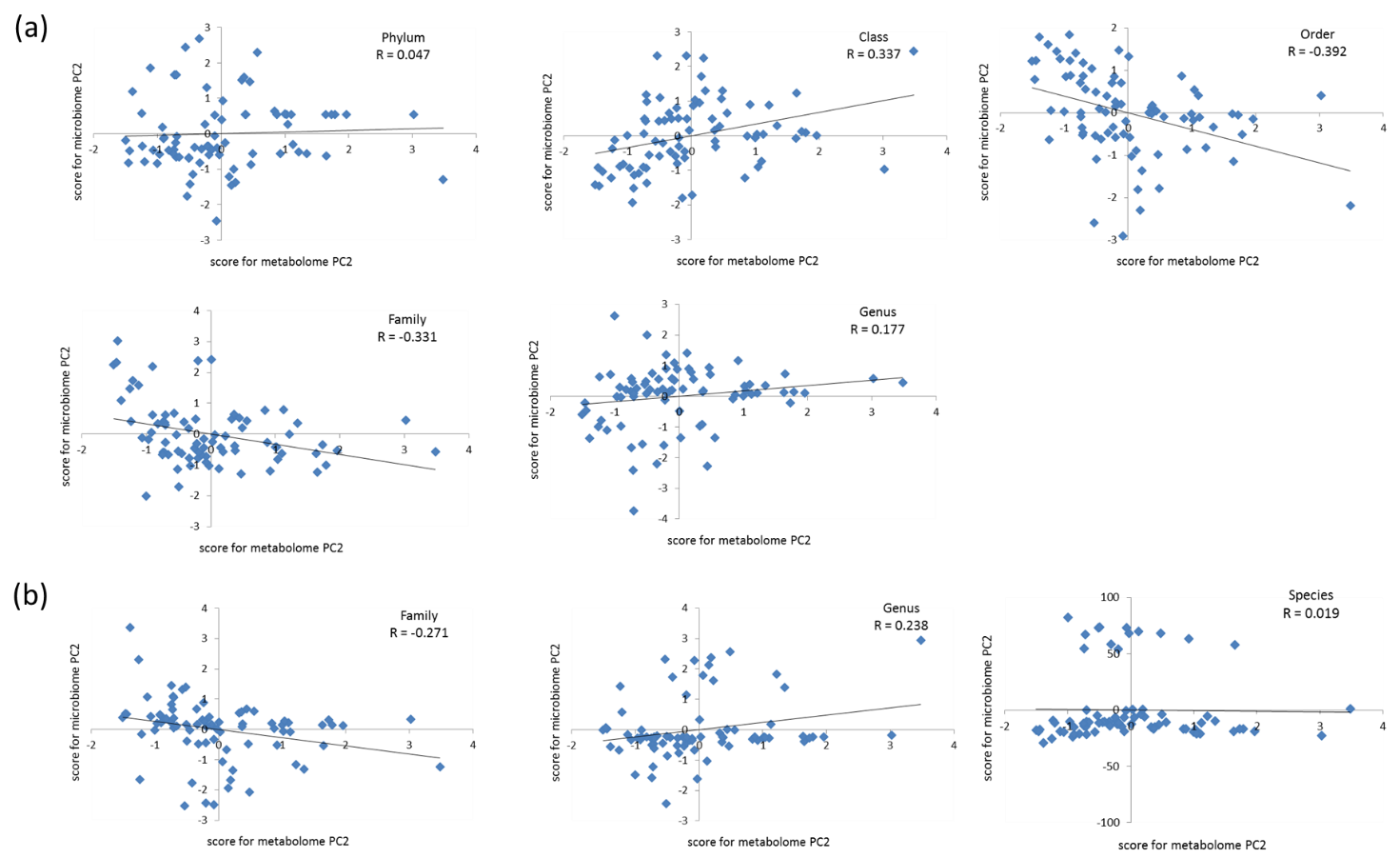


**Additional File 18. Correlation between score of PC2 of the metabolome and that of the microbiome**

(a) PC2 of classification data by RDP classifier. (b) PC2 of family-, genus-, and species-level classification data by OTU-based analysis. Scores for each principal component 2 (PC2) of the metabolome and microbiome were plotted in X-Y axis, followed by calculation of Pearson product-moment correlation coefficient.
